# HLHS: Power of the Chick Model

**DOI:** 10.3390/jcdd9040113

**Published:** 2022-04-11

**Authors:** David Sedmera

**Affiliations:** 1 Institute of Anatomy, First Faculty of Medicine, Charles University, 128 00 Prague, Czech Republic; David.Sedmera@lf1.cuni.cz; 2 Laboratory of Developmental Cardiology, Institute of Physiology, Czech Academy of Sciences, 142 20 Prague, Czech Republic

**Keywords:** left atrial ligation, left ventricular hypoplasia, hemodynamic alteration, myocyte proliferation, embryonic myocardium

## Abstract

Background: Hypoplastic left heart syndrome (HLHS) is a rare but deadly form of human congenital heart disease, most likely of diverse etiologies. Hemodynamic alterations such as those resulting from premature foramen ovale closure or aortic stenosis are among the possible pathways. Methods: The information gained from studies performed in the chick model of HLHS is reviewed. Altered hemodynamics leads to a decrease in myocyte proliferation causing hypoplasia of the left heart structures and their functional changes. Conclusions: Although the chick phenocopy of HLHS caused by left atrial ligation is certainly not representative of all the possible etiologies, it provides many useful hints regarding the plasticity of the genetically normal developing myocardium under altered hemodynamic loading leading to the HLHS phenotype, and even suggestions on some potential strategies for prenatal repair.

## 1. General Introduction

Hypoplastic left heart syndrome (HLHS) is a rare but deadly form of human congenital heart disease. Although its etiology remains unknown, there is an indication that it can be caused by genetic causes, prenatal infection, and hemodynamic alterations [1]. Since the genetic pedigrees indicate that HLHS is a part of a spectrum of diseases of the left heart structures, with the mildest form of hypoplasia of the left heart structure being the bicuspid aortic valve, there are currently no practically usable genetic models of HLHS available. To the contrary, experimental hemodynamic interventions have succeeded in creating HLHS in fetal lambs [2] and embryonic chicks [3,4,5,6]. Recently, a mouse fetal model of left heart hypoplasia was described [7] and is treated independently in another review of this Special Issue (https://www.mdpi.com/journal/jcdd/special_issues/HLHS, accessed on 28 March 2022). Since the chick embryonic model is more widely accessible, not least due to its cost and availability, the focus of this mini review is to discuss what was found in the chick experimental HLHS. These findings are put in perspective with human studies, where relevant.

## 2. Surgical Creation of the Chick Model

**Figure 1:**
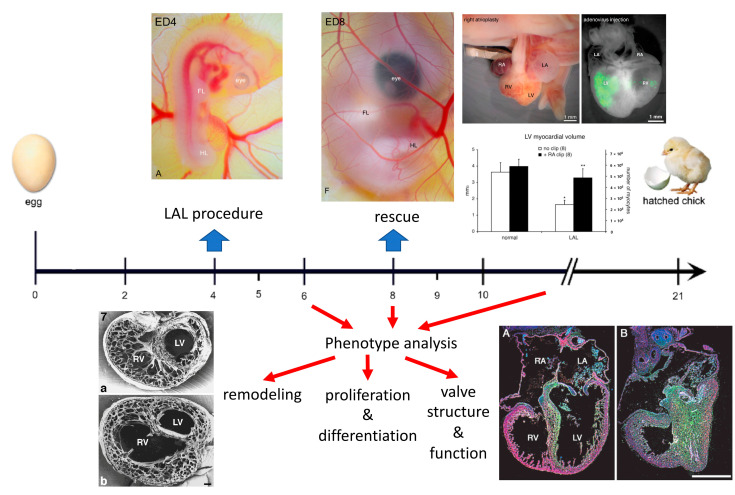
Scheme of the workflow for creating the chick model of HLHS and examples of phenotypic findings (below the time axis in days) and rescue attempts (top right). From references [1,6,8,9,10]. The time axis shows embryonic days; the heart starts to beat before ED2, develops trabeculae at ED3, completes ventricular septation at ED8, and completes functional coronary vasculature by ED9. The panels above the axis show the appearance of the chick embryo at ED4 and ED8 [8], rescued ED9 heart with a silver microclip on the right atrium, green fluorescence protein expression in the embryonic left ventricle confirming successful injection of the adenovirus [1], and the graph shows the increased myocardial volumes in the right atrial clip rescue experiment [9]. The bottom panels show the phenotype on transverse sections in the scanning electron microscopy 48 h after ligation (left) [6], and decreased myocyte proliferation demonstrated by increased retention of radiolabeled [3H]-thymidine [10] (green grains over the hypoplastic left ventricle).

In comparison to the 280 days of gestation for humans, chick development occurs within the timeframe of three weeks (Figure 1). The tubular heart starts beating during the second day of incubation, the chambers are formed by the third day, and the septation is completed by the eighth day of development.

Left atrial clipping or ligation (LAL, for procedure detail, see the Supplementary Materials) is performed at the preseptation stage and redirects the blood stream from left to right, thus resulting in the underdevelopment of left heart structures and compensatory overdevelopment of the right ventricle. The first studies by Rychter and associates [3,4] used a hand-forged silver microclip to restrict the volume of the developing left atrium by excluding the left atrial appendage (or auricle). They found that this procedure leads to overall decrease in mitotic counts, with numerous valve anomalies such as mitral atresia as a secondary consequence. A modification of this approach was presented by Harh and colleagues [5] by obstructing the left atrioventricular ostium with an insertion of a nylon thread; however, the main interest of these investigators was in the mechanisms of ventricular septation. Currently, the most popular (and technically easy) version developed by Norman Hu and associates is performed by ligating the portion of the left atrium by pre-prepared nylon loop [6]. It allows for control of the degree of perturbation, and usually, there is about 30% mortality up to the sampling a few days later, with about half of the embryonic heart showing the desired phenotype. Although this is not optimal, quantification is made possible by selection of only the hearts meeting the pre-set criteria (e.g., apex-forming right ventricle), resulting in decent statistical power. Typically, the LAL procedure is performed at embryonic day (ED) 4, and by completion of septation at ED8, the phenotype is distinct (Figure 1), although in more severe cases it is evident already after 48 h at ED6. The embryos can survive up to ED14 [6] and possibly later, and the phenotype tends to become more severe with time (decreasing LV:RV ratio, development of further complications such as mitral or aortic atresia) in a vicious circle [4]; however, the likelihood of mortality is increased in more severe phenotypes. This is similar to the human situation, where there is an ongoing argument whether the valvar changes are primary or secondary [1] and are treated more in depth in clinical papers of this Special Issue (https://www.mdpi.com/journal/jcdd/special_issues/HLHS, accessed on 28 March 2022).

## 3. Organ Level Changes Are Based upon Altered Myocyte Proliferation

On the morphological side, decreased volume loading accelerated morphogenesis of the volume-underloaded left ventricle, manifesting as precocious trabecular compaction at ED8 [6], which normally occurs between ED9 and 10 [11]. However, total myocardial volume was reduced, where the significant decrease in the left ventricle was not fully compensated by an increase in the right one. Increased volume loading in the right ventricle produced its dilatation, followed later by trabecular proliferation and thickening of the compact myocardium [6]. Thus, changes in wall thickness should be judged in perspective of total chamber size: the relatively collapsed left ventricle might appear to have a thicker wall (which, when quantified, is actually in the normal range), yet the dilated right ventricle might have, at the later stages, a thicker compact layer than the normal one. Changes in myocyte proliferation, documented in the early description [3] were studied in detail later using a label dilution approach [10]. Decreased proliferation in the left ventricular structure over time was evidenced by increased retention of radioactive label (^3^H-thymidine), administered prior to the procedure. In contrast, the 5-bromodeoxyuridine pulse labeling did not reveal any significant differences in any compartment. It was concluded that remodeling embryonic myocardial architecture in this model is based on the regulation of cell proliferation, as no differences were found in apoptosis using TUNEL staining. Fibroblast growth factor (FGF)-2 emerged as one possible candidate, which was found to be down-regulated in both left ventricular compact layer and trabeculae. In a follow-up study, the overexpression of FGF-2 by means of replication-deficient adenoviral vector [12] was able to increase myocyte proliferation by about 40% in both normal and HLHS left ventricular myocardium. It was also able to rescue contractile myocyte differentiation (as evidenced by anti-myosin immunostaining), which was shown to be decreased in the hypoplastic left ventricle [10]. These findings present one potentially feasible mechanism for prenatal intervention.

## 4. Prenatal Phenotypic Rescue

To verify the feasibility of rescue fetal surgical intervention(s) to reverse the development of HLHS, the chick model was employed at ED8. An ex ovo setup was used to monitor the acute hemodynamic effects of the reverse intervention (right atrial clipping), and echocardiography indeed showed increased volume loading of the hypoplastic ventricle [9]. Although such setups improve the access to the embryo by removing the egg shell, which was particularly useful for ultrasound biomicroscopy [13], it increases the risks of infection, water losses, and low calcium stores. Therefore, for extended survival, it was necessary to perform the clipping in ovo, which necessitated two operators and prior opening of the chest to gain access to the heart without procedural bleeding. Survival for 24 h was achieved, and at this sampling interval, both increased myocyte proliferation and increased myocardial volume were shown in double-operated hearts (i.e., LAL at ED4 plus clipping at ED8) in comparison to HLHS controls [9]. Significantly, no changes in right ventricular proliferation patterns were caused by the second intervention, yielding an experimental proof of principle for the success of human fetal catheter-based interventions [14,15,16,17].

The versatility of the chick model allows rapid testing of newly emerging technologies such as stem cells injection. For this purpose, it is advantageous to use either transgenic chick embryos expressing beta galactosidase [18] or GFP [19], or the time-tested chick-quail chimeras [20], to enable tracing of the donor cells. It was demonstrated that, although technically feasible, the injected embryonic bone marrow stem cells persisted, but did not contribute to the myocardium, forming blood islands and vascular structures (as could have been expected due to their origin and commitment) [1].

## 5. Functional Adaptation to Altered Hemodynamics

The function of the chick embryonic heart with developing HLHS was also studied to gain a better insight into myocardial adaptations to altered loading conditions. Wall deformation patterns were studied by Tobita and Keller [21]. LAL altered both right and left ventricular strain patterns, accelerating the onset of preferential right ventricular circumferential strain patterns, and abolished preferential LV longitudinal strain. This corresponded nicely with the altered trabecular orientation demonstrated previously by scanning electron microscopy [6]. At the cellular level, Schroder and colleagues studied myocardial strips of control and LAL hearts [22]. Two days after LAL, myocardial stresses at given strains and circumferential stiffness was increased in comparison to controls. Both total and polymerized beta-tubulin were increased in LAL samples, and confocal microscopy confirmed an increase in microtubule density in the LAL left ventricular compact layer. These changes were blocked by colchicine treatment. Passive myocardial properties were also analyzed by this group [23]. In the LAL hearts, the right ventricular end-diastolic volumes were increased, and the left ventricular ones decreased in comparison to controls two days after the procedure. These studies show that alterations in hemodynamic loading result in rapid myocardial remodeling, which is accompanied by differential changes in myocardial properties that persist over time, in contrast to adaptation to generally increased ventricular pressure load induced by outflow tract constriction [22,23] where the stresses and strains are normalized. Alterations in intracardiac blood flow LAL were also studied by Hu and associates [24]. They found a decrease in ventricular end-diastolic volume, and Indian ink injection showed right aortic flow patterns. The LAL thus disrupts both early cardiac morphogenesis and aortic arch selection.

The role of the hemodynamic pathway in the pathogenesis of HLHS is still actively studied using novel technologies such as high frequency ultrasound a computational fluid dynamics modeling [25]. The authors found, using 4D ultrasound imaging and cardiac flow simulation, that the atrial function was compromised, and flow velocity in the ventricle reduced, resulting in slower flow near the ventricular wall. They concluded that low and oscillatory flow near the left side of the heart could play a role in the development of HLHS in this model.

## 6. Alterations in Electrical Conduction and Fibrosis

Profound changes in myocardial architecture were expected to be accompanied by corresponding changes in conduction system development. Embryonic hemodynamic interventions manipulating the ventricular preload and afterload indeed resulted in changes in maturation of the cardiac conduction system [26,27]. Specifically, the maturation of the left bundle branch was inhibited in the settings of left ventricular hypoplasia [26], manifesting as a lack of an epicardial electrical activation site corresponding to the left bundle branch. Mechanistically, this was shown to be due to altered endothelin signaling [27] and validated by changes in expression of connexin40, a functional marker of rapidly conducting myocardium.

Although the chick model is a pathogenetic, rather than etiological, model of human HLHS, it brings up some interesting chicken or egg questions. It was used recently to probe the issue of endocardial fibroelastosis (EFE) [28], which commonly accompanies left ventricular hypoplasia, and by some, is also considered a possible etiology (prenatal viral endocarditis ending with fibrosis, which compromises left ventricular structure and could lead to its hypoplasia). An increased amount of subendocardial fibrous tissue containing collagen and periostin was demonstrated in older (ED12) left ventricles by immunohistochemistry. These findings were corroborated by mass spectrometry. Interestingly, these changes were preceded by an increased extent of the hypoxic regions, coinciding with the previously reported thickening of the left ventricular wall [6], pointing to yet another possible pathway leading ultimately to HLHS. However, the cellular composition of the hypoplastic left ventricular wall is entirely normal. Although the majority of cells at embryonic stages are myocytes, they are histologically more pale with a larger amount of extracellular space [6], and they express a lower amount of contractile proteins [10]. Consideration of other cell populations in the embryonic heart (such as endocardial cells, which influence the development of endocardial cushions that give rises to cardiac valves, impairment of which could also cause hemodynamic perturbations) should not be neglected.

## 7. Molecular Analysis and Comparison with Human Studies

An attempt at complete transcriptomic analysis of changes in both ventricles was performed by Krejci and associates [29] in the context of studying normal longitudinal development of the left and right ventricle. In general, most differentially expressed transcripts showed either delayed upregulation or downregulation. However, some extracellular matrix proteins such as fibulin were increased in the hypoplastic left ventricle, whereas some proteins associated with proliferation (cyclin K) were decreased. Unfortunately, the state of gene annotation precluded more detailed analysis that would allow comparison with a recent human transcriptomic study [30] that clearly showed that one possible pathway to HLHS could be an intrinsic myocyte defect resulting in their premature exit from cell proliferation. How this could be potentially reversed by mechanical factors (such as fetal or neonatal surgical restoration of normal hemodynamic loading) remains to be tested.

In conclusion, the power of the chick model of HLHS lies in its simplicity, ability to control the level of ligation, and to some degree, its timing. The annotation of the chick genome improves continuously, making it useful also for molecular and mechanistic studies [31], and it is also a feasible model for testing potential prenatal therapeutic interventions [9,12]. The main limitation is that the chick model of HLHS is not genetically based, although in some human genetic defects, the mutations may primarily affect the cardiac cushions, making the myocardial defects also secondary. Genetic origins of human HLHS are explored in detail by several other reviews in this Special Issue (https://www.mdpi.com/journal/jcdd/special_issues/HLHS, accessed on 28 March 2022). Although the organism is homeothermic and the heart fully septated, it is still a non-mammalian organism, so any extrapolation to humans requires extra caution.

## Data Availability

Not applicable.

## Supplementary Materials

The following supporting information can be downloaded at: https://www.mdpi.com/article/10.3390/jcdd9040113/s1, Video S1 (MVI_3387_LAL.AVI): Video of left atrial ligation (LAL). The embryo is first flipped to the left side up position, and afterwards, returned to the original right side up position (not shown in the clip).
